# Breathlessness, but not cough, suggests chronic obstructive pulmonary disease in elderly smokers with stable heart failure

**DOI:** 10.1186/s40248-018-0148-1

**Published:** 2018-10-01

**Authors:** Sara Roversi, Piera Boschetto, Bianca Beghe’, Michela Schito, Martina Garofalo, Mariarita Stendardo, Valentina Ruggieri, Roberto Tonelli, Alessandro Fucili, Roberto D’Amico, Federico Banchelli, Leonardo M. Fabbri, Enrico M. Clini

**Affiliations:** 1 Department of Medical and Surgical Sciences, University of Modena and Reggio Emilia, Modena, Italy; 2 Department of Medical Sciences, University of Ferrara, Ferrara, Italy; 3 Department of Respiratory Diseases, University of Modena and Reggio Emilia, Modena, Italy; 4 Department of Cardiology, and LTTA Centre, University-Hospital of Ferrara, Ferrara, Italy; 5 Statistic Unit, Department of Diagnostics, Clinical and Public Health Medicine, University of Modena and Reggio Emilia, Modena, Italy; 6 Sahlgresnska University-Hospital, Gothenburg, Sweden

## Abstract

Chronic obstructive pulmonary disease (COPD) is a common comorbidity of heart failure (HF), but remains often undiagnosed, and we aimed to identify symptoms predicting COPD in HF. As part of an observational, prospective study, we investigated stable smokers with a confirmed diagnosis of HF, using the 8-item COPD-Assessment-Test (CAT) questionnaire to assess symptoms. All the items were correlated with the presence of COPD, and logistic regression models were used to identify independent predictors. 96 HF patients were included, aged 74, 33% with COPD. Patients with HF and COPD were more symptomatic, but only breathlessness when walking up a hill was an independent predictor of COPD (odds ratio = 1.33, *p* = 0.0484). Interestingly, COPD-specific symptoms such as cough and phlegm were not significant. Thus, in elderly smokers with stable HF, significant breathlessness when walking up a hill is most indicative of associated COPD, and may indicate the need for further lung function evaluation.

## Background

The interaction between the lung and the heart is a complex, vast, and fascinating subject, and from a clinical point of view, disorders of the one often influence and promote disorders of the other. This is especially true for chronic diseases, with heart failure (HF) and chronic obstructive pulmonary disease (COPD) representing a common example: although prevalence rates vary across published literature, it is estimated that about 17–35% of patients with HF are affected by COPD [1]. Despite having important diagnostic, prognostic, and therapeutic implications [2, 3], this disease seems to be rather underestimated, and a significant rate of HF patients with associated COPD remains undiagnosed and untreated [4]. It is known that the coexistence of these two disorders poses different challenges to the treating physician (e.g., poor echocardiographic acoustic windows, putative negative effects of bronchodilators, supposed limitation in beta-blocker use) [5], but it should not be forgotten that the first challenge is making a correct diagnosis in the patient with HF and persistent dyspnea: is it because of the heart or the lung?

In this regard, a careful assessment of symptoms is the first approach for making a correct diagnosis (although additional investigations such as spirometry are eventually required) [6]. The purpose of our study was to evaluate the clinical presentation of stable smokers with an establish diagnosis of HF, measuring their symptoms with questionnaires and aiming to identify independent clinical predictors of associated COPD. We had a pragmatic approach, using quick and simple tools that could easily be applied in everyday practice.

## Methods

The present study is part of an observational, prospective, 3-year study conducted in a population of stable elderly smokers[NCT01893918]. Subjects included in the present investigation were consecutively enrolled while attending regular follow up visits at a specialized outpatients clinic for HF, all being subjects with a diagnosis of HF confirmed by a cardiologist team, including ischemic and non-ischemic HF, as well as reduced and preserved ejection fraction. To be evaluated for enrollment, the following criteria were required: aged ≥65 years; smoking history of ≥20 pack/years; clinical stability. Exclusion criteria included: other pulmonary conditions, cancer, acute inflammatory diseases: new diagnosis of a major cardiac comorbidity within the last 6 months; worsening of symptoms in the last 6 months; inability to comply with study and/or informed consent. The study conformed to the Declaration of Helsinki and was approved by the institutional ethics committees. All participants provided informed consent before recruitment.

After enrollment, all participants underwent several clinical investigations, including recording of demographic data, basic biochemistry, echocardiography, and screening for COPD with symptom assessment and spirometry. Spirometric diagnosis of COPD was confirmed according to the postbronchodilator forced expiratory volume in the 1^st^ second/forced vital capacity (FEV_1_/FVC) ratio < 0.7. [6] Symptoms were evaluated in all patients using both the modified Medical Research Council (mMRC) dyspnea scale and the COPD-Assessment-Test (CAT), which have been first developed for COPD, [7] and later used in patients with concomitant COPD and HF [8, 9]. The mMRC grades the level of dyspnea on a scale from 0 to 4, while the CAT evaluates 8 different aspects of patient life (1-cough, 2-phlegm, 3-chest tightness, 4-breathlessness, 5-limitations doing any activities at home, 6-confidence in leaving home, 7-sleep and 8-overall energy) grading each one on a 0–5 scale. For both tests, higher scores indicate worse symptoms. Finally, study investigators (including cardiologists and pulmonologists) established a diagnosis of HF alone or HF and COPD, based on all available study data.

For the current study, baseline symptom assessment was correlated with the final diagnosis (HF alone vs. HF and COPD). The mMRC and the CAT items were used as standardized descriptors of patients’ symptoms, providing a uniform and measurable assessment in all patients. Descriptive statistics were used to compare baseline characteristics between the two groups of HF or HF and COPD, while univariate and multivariate logistic regression models were used to search for independent predictors of COPD. Analyses were performed by means of R 3.3.2 statistical software (The R Foundation for Statistical Computing).

## Results

Study population included a total of 96 patients with HF, of whom 32 (33%) had a final diagnosis of concurrent COPD. Patients were mostly male, aged 74 ± 5.6, and either current or former smokers with mean 39 ± 20 pack/years. Left ventricle ejection fraction ranged 21 to 66%, although on average was only mildly depressed (44% ± 10). Not surprisingly, comorbidities were frequent, including ischemic heart disease and diabetes. Overall, baseline characteristics did not differ significantly between patients with or without COPD (Table 1).

**Table 1:** Characteristics of the population, overall and group-specific

	Overall (*n* = 96)	HF (*n* = 64)	HF + COPD (*n* = 32)	p*
Age – mean (SD)	74.14 (5.6)	74.14 (5.6)	74.13 (5.7)	.99
Male (%)	91	89	94	.45
Pack/year – mean (SD)	39.1 (20.0)	39.0 (20.0)	39.4 (20.5)	.93
BMI – mean (SD)	28.7 (4.3)	28.8 (3.7)	28.6 (5.3)	.83
6MWT – mean (SD)	397 (160)	419 (155)	353 (164)	.06
LVEF– mean (SD)	43.8 (10.0)	44.6 (10.0)	42.0 (10.2)	.24
Hb – mean (SD)	13.6 (1.7)	13.6 (1.8)	13.6 (1.6)	.98
GFR – mean (SD)	56.5 (20.1)	56.7 (19.9)	56.2 (20.8)	.90
Comorbidities				
CCI – mean (SD)	5.9 (1.7)	5.8 (1.6)	6.1 (1.8)	.43
IHD (%)	65.6	67	62	.65
Stroke (%)	3.1	1.6	6.2	.21
CKD (%)	12.5	10.9	15.6	.51
PAD (%)	11.5	10.9	12.5	.82
Met synd (%)	67.7	71.9	59.4	.21
Diabetes (%)	34.4	39.1	25.0	.17
Osteoporosis (%)	2.1	1.6	3.1	.61
Anxiety (%)	4.2	1.6	9.4	.07
Symptoms				
mMRC – mean (SD)	1.3 (0.95)	1.1 (0.92)	1.72 (0.9)	.001
CAT – mean (SD)	9.9 (6.2)	9.4 (6.3)	11.0 (6.0)	.001

*comparison between patients with HF and patients with HF + COPD

6MWT: 6-min walk test, *BMI* body mass index, *CAT* COPD assessment test, *CCI* Charlson comorbidity index, *CKD* chronic kidney disease, at least moderate, *GFR* glomerular filtration rate, *Hb* hemoglobin, *IHD* ischemic heart disease, *LVEF* left ventricle ejection fraction; Met synd: metabolic syndrome, *mMRC* modified medical research council, *PAD* peripheral artery disease

**Fig. 1:**
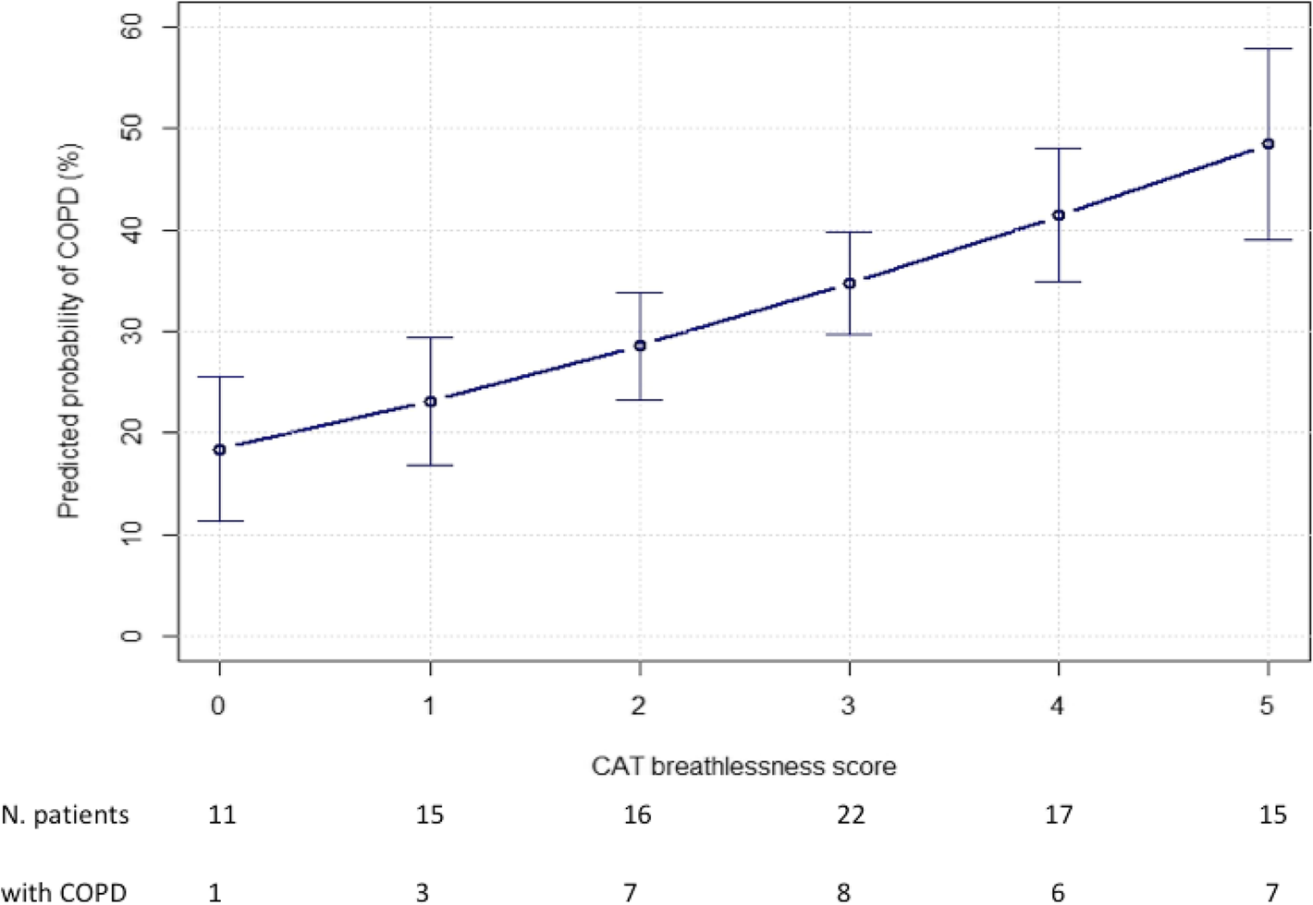
Patient's probability of havig COPD according to each 1-point increase in CAT score

Enrolled HF patients were on average only mildly symptomatic, with a mean mMRC of 1.3 ± 0.9 and CAT of 9.9 ± 6.2. Both scores were significantly higher in patients with HF and COPD, as compared to HF alone (1.7 ± 0.9 vs. 1.1 ± 0.9; *p* = 0.001, and 11.0 ± 6.0 vs. 9.4 ± 6.5;*p* < 0.001). Despite reaching statistical significance, the absolute difference in mMRC was too small to find clinical application, and further analysis was not performed. On the contrary, each item of the CAT score was evaluated singularly, with cough, phlegm, and breathlessness being more frequent in patients with both diseases. However, according to our multivariate logistic regression model, after adjustment for sex, age and smoke exposure, only item n.4 (breathlessness when walking up a hill or one flight of stairs) maintained a linear correlation with the presence of COPD: for a 1-point increase in the score, there was a relevant and statistically significant increased risk of being diagnosed with concomitant COPD [odds ratio (OR) 1.33 (95% confidence interval(CI) = 1.00–1.77; *p* = 0.048)], (Fig. 1).

## Discussion

The results of our study could be summarized in a single, practical indication: when evaluating a smoker, elderly man with stable HF, ask her/him to grade her/his breathless when walking up a hill or one flight of stairs, from 0 (absent) to 5 (very breathless). Her/his answer will give a simple and quick estimate of the risk of having concomitant COPD, and in patients with high risk further testing may be indicated. By contrast, and rather unexpectedly, cough and sputum do not seem as helpful in suggesting high risk of having concomitant COPD.

It is known that the identification of COPD in patients with HF represents a challenge. Since the fundamental symptom of one disease, i.e. dyspnea, is the leading symptom also of the other, it is not uncommon to mistake one for the other. Thus, the rate of underdiagnosis of COPD in HF may be as high as one every three patients [10]. Nevertheless, previous studies investigating clinical characteristics of patients with established diagnosis of COPD and HF have reported significant differences, such as higher New York Heart Association class, higher prevalence of dyspnea at rest, lower functional status, and lower quality of life in patients with both conditions [11, 12].

Thus, we believed that a pragmatic assessment of symptoms in stable, elderly smokers with known HF could provide a quick and useful first tool to evaluate the risk of associated COPD, and therefore prompt further evaluation of lung function. By using a standardized questionnaire such as CAT, we could objectively measure symptoms in our population, and highlight the most significant ones. According to our result, higher grade of breathlessness is the best indicator of concomitant COPD, which is in line with previous data. [13] As shown in Fig. 1, the probability to find concomitant airway obstruction increases progressively and linearly with increasing breathlessness, from 18.4 to 48.5%. Interestingly, symptoms such as cough and phlegm were different between patients with HF and patients with HF and COPD at univariate analysis, but did not maintain a correlation at multivariate analysis. This is probably explained by the low prevalence of cough and sputum in our population, suggesting that these symptoms are indeed disease-specific, [14] but are not frequently reported by stable patients. Thus, although cough with or without phlegm is one of the principal symptoms of COPD, [15] clinicians should not rely only on it to suspect this lung disorders.

Our study presents some limitations, with the small sample size being the major one. Moreover, the patients were overall only mildly symptomatic, thus masking potential differences among groups. Furthermore, there were some baseline differences, such as higher prevalence of anxiety in patients with both COPD and HF. Although not being significantly different, and thus difficult to further analyze, such differences may have influenced the perception of breathlessness. Similarly, the use of a fixed ratio for COPD, although recommended by the latest GOLD document as the diagnostic criterion for airflow limitation, [6] has been shown to overestimate the diagnosis in the elderly, which may have affected the results. Therefore, interpretation of these data is not univocal and definite conclusion cannot be drawn. Thus the study itself should be regarded as hypothesis generating. Nevertheless, being an observational study enrolling consecutive patients from a cardiologic clinic, our study is probably fairly representative of everyday clinical practice. Additional studies are needed to replicate our findings in a larger population, and to evaluate their effective utility.

## Conclusion

To conclude, in the era of technological medicine, these data remind us of the importance of measuring patients’ symptoms. Grading breathlessness may point clinicians in the right direction when evaluating stable, smoking patients with HF, i.e. suspecting the presence of lung impairment and help uncovering COPD. Our findings remain mostly speculative and hypothesis-generating, and future studies are needed to confirm these preliminary data, and to evaluate their cost-effectiveness in everyday practice.

## Abbreviations

CAT: COPD-Assessment-Test (CAT)
COPD: Chronic obstructive pulmonary disease
HF: Hearth failure
mMRC: Medical Research Council (mMRC) dyspnea scale
OR: Odds ratio
